# Evidence-Based Policy Recommendations for Public Health Emergency Operation Centers in Regional Settings: A Case Study in Indonesia

**DOI:** 10.3389/phrs.2023.1604899

**Published:** 2023-08-04

**Authors:** Muhammad Hardhantyo, Hanevi Djasri, Aldilas Achmad Nursetyo, Bella Donna, Madelina Ariani, Happy Pangaribuan, Gde Yulian Yogadhita, Andriani Yulianti, Bernadeta Rachela Adipradipta

**Affiliations:** ^1^ Center for Health Policy and Management, Faculty of Medicine, Public Health and Nursing, Universitas Gadjah Mada, Yogyakarta, Indonesia; ^2^ Faculty of Health Science, Universitas Respati Yogyakarta, Yogyakarta, Indonesia

**Keywords:** Indonesia, PHEOC model, disease outbreak, COVID-19, management

## Abstract

**Background:** Public health emergencies require integration between multiple stakeholders in different sectors to monitor the situation and carry out an appropriate response. As a country with a large land area consisting of thousands of islands, Indonesia’s centralized Public Health Emergency Operation Center (PHEOC) system is currently unable to effectively contain diseases. A PHEOC system reform is required to accommodate Indonesia’s circumstances, particularly at the regional level. We have outlined potential models at the sub-national level for PHEOC based on existing evidence.

**Policy Options and Recommendations:** Based on existing evidence of PHEOC models internationally, we have formulated three policy models for regional-level PHEOC. These models (the *ad hoc* agency model, the independent agency model, and the Province Health Office (PHO)-based model) entail different chains of command, and each has its own benefits.

**Conclusion:** We recommend that the Ministry of Health in Indonesia adopt the third PHEOC policy model, in which the chain of command lies under the PHO. This is the most practical approach, as the PHO has the authority to mobilize units and access resources in response to imminent public health emergencies. Further training and capacity-building are required to support the PHO as the commander of the regional PHEOC.

## Background

Large-scale public health emergencies demand close coordination across multiple sectors to address urgent health needs and support the affected population. The World Health Organization (WHO) recommends the implementation of Public Health Emergency Operation Centers (PHEOC) as a critical framework to coordinate stakeholders, collect information, and allocate resources during public health emergencies [1]. This framework integrates public health services and related entities into a single emergency management team that can operate at national and regional levels. While the implementation may vary within each country, WHO has released guidelines for PHEOC development to ensure consistency and effectiveness.

Indonesia reported its first COVID-19 case in March 2020, and 1 year later, daily confirmed cases reached 50,000. However, Indonesia’s government declared a public health emergency only 3 weeks after the first case, which many argue was too late to effectively control the virus’s spread [2, 3]. In contrast, New Zealand’s government detected its first COVID-19 case 1 week before Indonesia’s, and community transmission occurred 2 weeks later. Despite lacking sufficient capacity for testing and contact tracing, New Zealand quickly shifted its focus from mitigation to an elimination strategy, resulting in a stricter nationwide lockdown policy [4]. After 7 weeks of lockdown, community transmission was stopped, and New Zealand ended its pandemic status after 103 days. New Zealand’s government effectively communicated its pandemic status and strategy to the public [5]. Nigeria also successfully implemented “containment strategies” learned from Ebola transmission control in 2014. Nigeria invested in PHEOC development with guidance from the United States Centers for Disease Control and Prevention [6].

The spike in Indonesia’s pandemic curve highlights a gap in the effectiveness of the current national PHEOC model. At the regional level, the public health emergency was managed by a surveillance division in the Province Health Office (PHO) and the Province Disaster Management Agency (BPBD) as an *ad hoc* team without a clear division of responsibilities [7]. Adapting best practice guidance suited to local circumstances could incentivize the full implementation of outbreak prevention, early detection, and response strategies. Identifying and correcting deficiencies in evaluating effectiveness can provide the basis for continuous PHEOC improvement [8]. In this policy brief, we outline the potential of adopting PHEOC at a sub-national level based on existing evidence to improve regional capacity in response to public health emergencies.

## Evidence

During the COVID-19 pandemic, the Indonesia government established a national *ad hoc* team called *Gugus Tugas COVID-19* (COVID-19 Task Force), led by the National Agency for Disaster Management. While the agency was trained to handle natural disasters, they lacked experience in managing public health crises. The team’s primary responsibility was to coordinate and mobilize various resources to control the spread of COVID-19. While the PHEOC team within the Ministry of Health provided advisory support to the COVID-19 Task Force, its effectiveness in responding to the outbreak was limited due to its focus on epidemiological data collection, monitoring, and reporting. The team lacked the operational capability to effectively coordinate and mobilize resources during an outbreak, which is essential to implementing timely and effective response measures. This highlights the need for improved coordination and collaboration between different government agencies and stakeholders, as well as a strengthened public health emergency management system that can respond quickly and effectively to future pandemics and public health emergencies [9].

At the regional level, the management of all resources related to disease outbreak response falls under the purview of provincial governments, which include the Public Health Office (PHO) and Regional Agency for Disaster Management (RADM). According to government regulation, the RADM are responsible for conducting situational analysis, managing resources, and developing policies [10]. However, recent studies suggest that provincial governments have failed to make timely decisions and mobilize resources to improve testing, contact tracing, and transmission prevention. This has been attributed to a lack of experience in managing public health emergencies, a complex bureaucracy, inconsistent rules, a lack of policy synchronization with the national government, and community distrust [11].

The current situation of the National PHEOC in Indonesia highlights an incapability to effectively manage a public health emergency response at the regional level across the country’s 37 provinces. During emergency responses, timely decision-making based on accurate and comprehensive information is crucial for effective management of the situation. Historical evidence of well-handled disease outbreaks emphasizes the importance of this approach. It is essential to ensure that all teams involved in emergency responses have the necessary skills, resources, and support to respond quickly and effectively [12]. In order to improve the capability of the National PHEOC, there is a need for better collaboration and coordination between different government agencies and stakeholders, as well as capacity-building initiatives that focus on strengthening the emergency response system and providing training for key personnel involved in managing public health emergencies.

According to current regulations, PHEOC teams are expected to respond within 24 h of receiving alerts of a potential disease outbreak, followed by an epidemiological investigation within 48 h of suspected case discoveries. Laboratory specimens should be sent within 48 h of collection, and results from the laboratory should be reported to the PHEOC within 24 h [7, 13]. In November 2017, Indonesia faced a diphtheria outbreak, and the Ministry of Health responded by launching an Outbreak Response Immunization campaign targeting children under 18 on December 11th of that year [14]. Six months after a three-dose diphtheria ORI was conducted in East Java, there was a significant decrease in diphtheria cases [15]. However, in 2005, Indonesia experienced a fatal human H5N1 outbreak, which was attributed to a failure to contain a poultry outbreak that had occurred 2 years prior [16].

An intra-action review conducted 8 months after the first COVID-19 case was detected in Indonesia revealed significant shortcomings in the country’s early detection and response capabilities. Despite previous experience with emerging infectious disease threats such as avian influenza (H5N1 & H1N1) and MERS-Cov, Indonesia struggled with command coordination, operational support, and logistics during the COVID-19 outbreak. This was largely due to the country’s decentralized government system, which limited the regional capacity to adopt and implement national policies [17]. Furthermore, it was found that Indonesia has limitations in laboratory diagnostics despite having laboratory networks for influenza diagnosis due to past avian influenza outbreaks [18]. These reports suggest that the current PHEOC model lacks coordination between the national and regional government, resulting in ineffective outbreak detection and response in Indonesia.

The examples of Uganda and Malaysia highlight the benefits of having a single centralized national entity, such as PHEOC, that can be quickly activated in times of emergency. In Uganda, the central government makes prompt decisions to activate the PHEOC and establish both National and District Task Forces during Ebola outbreaks, which mobilize resources and coordinate the full range of Ebola preparedness plans. In Malaysia, the PHEOC is also activated immediately in response to public health emergencies, and is responsible for coordinating response efforts, conducting risk assessments, and managing resources. These centralized models have proven effective in enabling a timely and coordinated response to public health emergencies, which can be critical in containing and preventing the spread of infectious diseases [19]. In 2020, during the COVID-19 pandemic, the CPRC played a critical role in managing the outbreak. The agency was responsible for coordinating and communicating between various stakeholders including the Ministry of Health, other government agencies, and the public. The CPRC acted as the central command center for the national response, providing real-time updates on the situation, advising the government on policy decisions, and coordinating resource mobilization across the country. The success of Malaysia’s response to the COVID-19 pandemic can be partly attributed to the centralized and coordinated approach of the CPRC [20, 21]. The PHEOC model in Indonesia is quite different from the centralized model in Uganda and Malaysia. In Indonesia, the responsibility for managing public health emergencies is shared between the national and regional governments, which can create challenges in terms of coordination and resource mobilization. Additionally, the current PHEOC model in Indonesia has been criticized for its lack of effectiveness in outbreak detection and response, which suggests that there is room for improvement in the country’s approach to managing public health emergencies.

The Regional PHEOCs in Vietnam are responsible for detecting and responding to public health emergencies at the regional level. They have the authority to coordinate with the central government and local authorities to mobilize resources and implement emergency response plans. The Regional PHEOCs also collaborate with the national PHEOC to ensure that there is a synchronized response across the country. This decentralized model allows for a more regionally-tailored response to public health emergencies, taking into account the specific needs and resources of each region [22]. The PHEOC in Vietnam regularly conducts and coordinates training sessions for Ministry of Health and regional personnel involved in public health emergencies, as well as conducting multiple drills and tabletop exercises to prepare for potential outbreaks [23]. It is true that implementing a decentralized PHEOC model similar to Vietnam’s may pose challenges in Indonesia due to its vast geographic size and numerous islands. The cost of building and maintaining dedicated PHEOC buildings with ICT infrastructure and technical staff in each province may also be a major hurdle. Additionally, ensuring policy synchronization and coordination between the national and regional governments may be difficult. Therefore, Indonesia may need to explore other models that can effectively address its unique situation while still ensuring timely and coordinated responses to public health emergencies [24]. This model acts as an independent body and becomes the focal point to manage public health emergencies.

The Nigerian PHEOC model is a decentralized one, with each state having its own PHEOC to manage public health emergencies. The PHEOC is integrated into the Ministry of Health’s government-owned building in each state and staffed with personnel trained in public health emergency management. This model allows for better coordination between the national and regional levels and provides a more localized response to public health emergencies [25]. In Indonesia, there are currently initiatives aimed at improving the capacity of regional PHEOCs due to their minimal infrastructure, undocumented operational activities, and lack of clear responsibilities among staff [26]. The staff will remain active throughout the year as they are also employed as epidemiologists working in the Provincial Health Office. They conduct routine surveillance during both outbreak and non-outbreak periods. The trained staff are capable of responding to alerts from the Early Warning Alert and Response System within 24 h [27, 28]. Since the training, there have been coordinated responses to four outbreaks in South Sulawesi Province, including COVID-19, rabies, diphtheria, and currently monkeypox [29–31]. Based on surveillance data in South Sulawesi Province, 96% of potential outbreak disease alerts have been addressed within 24 h following the training, which represents a significant improvement compared to previous years where the figure was as low as 81%. This increase is mainly attributed to the greater awareness among the surveillance team about the importance of early reporting and the potential impact of outbreak events [32].

One PHEOC model that has proven effective is integrated directly into the existing health-related regional government office, allowing for better preparation, collaboration, and response during public health emergencies. In this model, authority is granted to the Provincial Health Office (PHO) to function as the focal point of coordination and communication, provide analysis, and monitor outbreaks. Its physical location in the multifunction room in the Province Health Office makes it easier for teams to plan and work together [28]. This model is a basic and cost-effective PHEOC that is able to respond to any local or regional public health emergency. It provides an essential platform for the management of public health emergencies, helping to avoid delayed decision-making, poor coordination, and over- or under-use of expertise and special resources.

This evidence demonstrates the need for additional support from various sources to enable regional health systems to respond immediately, prevent leadership failures, and recover to be better equipped to handle future public health emergencies. Public health emergency situations demand innovation and deviation from conventional approaches. Routine health services may be overwhelmed and unable to operate efficiently during emergencies. Moreover, while the guidelines for operationalizing the national PHEOC are clearly established, the challenges of implementing PHEOC at the regional or provincial level are more intricate [33].

Indonesia’s government must go beyond its fragmented and reactive approach to develop a clear and cohesive strategy for addressing public health crises. This can be achieved by establishing local and regional PHEOCs that can swiftly coordinate information and resources. However, establishing effective PHEOCs relies on the availability of appropriate infrastructure, workflow, human resources, plans, procedures, and command structures [34].

## Policy Options and Recommendations

Indonesia’s regional PHEOCs require emergency management systems that are integrated with daily routine preparedness systems in each province. The organizational models presented below offer three examples along with descriptions of the communication integration and coordination among sectors within the health office, or across sectors when dealing with disasters and health crises. These models can be used as a reference to consider the sustainability of PHEOCs in the post-pandemic era or to improve the existing PHEOCs in Indonesia.

### Model 1

Based on the evidence presented above, we have formulated three policy options that could be adopted by Indonesia’s regional health system, as described in Table 1. The table shows three different models. Model 1, which is currently employed in the Indonesian PHEOC handling of the COVID-19 pandemic, places the PHEOC outside of the Health Office and under the Government as an *ad hoc* team (i.e., BPBD, COVID-19 task force). Centralized command from district/provincial leaders makes it easy to mobilize resources. However, in this model, success is highly dependent on the leadership quality of the regional head, and sustainability in post-disaster situations is constrained by limited funding for *ad hoc* teams, which only work for short periods during a given situation. As a result, the team is unable to continue its work in post-emergency situations. Since Model 1 is only in the form of an *ad hoc* team, synergies and interoperability are necessary between *ad hoc* institutions and the local health authorities to receive routine surveillance data, mobilize resources during outbreaks, and in non-outbreak situations.

**TABLE 1:** Policy options for the Public Health Emergency Operation Center Model Implementation in Indonesia (Building Capacity for the Regional Public Health Emergency Operations Center in South Sulawesi Province, Makassar City, and Maros Regency, Indonesia, 2021).

	Advantage	Disadvantage
Model 1		
Centralized PHEOC and regional PHEOC form an *ad hoc* team (i.e., BPBD, COVID-19 task force)	1. Supported with strong regulation to mobilize resources during surge capacity	1. Complex bureaucracy makes rapid decision-making difficult in large nations, i.e., Indonesia
	2. *Ad hoc* team consists of expert specialists in the field	2. Unsynchronized policy between national government and provincial government
	3. Leader can be adjusted according to public health emergency	3. Limited time for *ad hoc* team to respond the public health emergency
		4. Continuity funding is still questionable during the non-outbreak situation
		5. Interoperability with the Province Health Office is needed to receive routine surveillance data
Model 2		
Decentralized PHEOC outside the government system	1. Trained staff capable of managing public health emergency during outbreak and non-outbreak period 2. Physical location makes it easier to collaborate, plan, and implement emergency responses	1. Large investment needed to establish physical location and hire dedicated staff for all regional PHEOC in the country
		2. Limited regulation for non-governmental organization to mobilize resources, manage logistics
		3. Interoperability with government organization is needed to receive routine surveillance data
Model 3		
PHEOC physically located in Province Health Office	1. Low investment to establish regional PHEOC	1. There is a need for routine training for the Province Health Office's staff to address public health emergency situations
	2. Staff remain active through the year, and know their duties and roles during outbreak and non-outbreak situations	2. Staff may have an additional work and be occupied with routine activities during the non-outbreak situation
	3. Team has authority to mobilize units and access resources in the provincial area in response to imminent public health emergency	
	4. Located inside the province, enabling rapid response to local public health emergencies and facilitating team planning and collaboration	

BPBD, Province Disaster Management Agency.

### Model 2

In Model 2, the regional PHEOC functions outside the government system. The organization is usually attached to a university or stands as an independent institution. Professionals trained in disaster management can work quickly in the pre-disaster and disaster phases. However, applying this model at the provincial level will require additional resources, particularly for dedicated staff and a suitable location. This model might work best in a small country. Adapting it in Indonesia will prove difficult because of budget constraints and the need for the largest investment in PHEOC infrastructure outside the government system. Existing regulations do not yet provide support for non-governmental organizations to become resource mobilizers and only allow them to take on the role of coordinators without a command role.

### Model 3

Model 3 places the PHEOC within the Province Health Office. This model utilizes the capacity of local health resources, which contribute to improved mitigation and response systems. This model is easier to implement than others due to the authority of the Province Health Office to manage resources and develop a fast tactical response in their area. However, it depends on an increased capacity and awareness of handling public health emergencies. During a potential public health emergency detected by the surveillance unit, the PHEOC is activated, and the officers at the health office must already know their roles and responsibilities to respond to the alert.

We suggest that the Ministry of Health adopt the third model (PHEOC command under Province Health Office) since it is the most practical form of PHEOC and can be deployed in most provinces in Indonesia. Routine drills and disaster management training are mandated by Indonesia’s MoH Regulation No. 13 of 2022, in the form of tabletop exercises, case simulations, or developing contingency plans. This model can improve the timeliness of decision-making during public health disasters, both nationally and regionally, as outlined by academics and policy researchers [3, 35].

## Conclusion

Among the policy options for implementing PHEOC in regional settings, the third model of PHEOC under the Province Health Office is the most suitable for implementation in Indonesia. Each province and district in Indonesia has a PHO backed by regulation and human resources. It has the capability to mobilize resources required to respond to health emergencies. The PHO should be supported by a health contingency plan to help measure the degree of disaster and respond appropriately by mobilizing available resources in the region. Indonesia’s MoH Regulation No. 75 of 2019 on Health Crisis Countermeasures should be adjusted to accommodate regional PHEOC coordination to strengthen the PHO position in responding to public health disasters.

## Abbreviations

PHEOC, Public Health Emergency Operation Center; PHEIC, Public Health Emergency of International Concern; CDC, Centers for Disease Control and Prevention; WHO, World Health Organization; MoH, Ministry of Health; BPBD, Province Disaster Management Agency; CPRC, Crisis Preparedness and Response Centre.
